# HPV testing in cervical cancer formalin-fixed paraffin embedded tissues: Reliability of the Xpert HPV test and high-risk HPV genotypes distribution in Tunisia

**DOI:** 10.1371/journal.pone.0333600

**Published:** 2025-10-24

**Authors:** Yasmine Ben Taher, Mariem Ben Rekaya, Emna Romdhane, Baligh Miladi, Linda Bel Hadj Kacem, Ahmed Med H’mayada, Nadia Ben Mansour, Farah Sassi, Emna Chelbi, Monia Tangour, Ahlem Blel, Soumaya Rammeh

**Affiliations:** 1 Faculty of Medicine of Tunis, LR23ES02, University Tunis El Manar, Tunis, Tunisia; 2 Pathology Department, Charles Nicolle Hospital, Tunis, Tunisia; 3 Epidemiology and Preventive Medicine Department, Charles Nicolle Hospital, Tunis, Tunisia; 4 Pathology Department, Taher Maamouri Hospital, Nabeul, Tunisia; 5 Histopathology laboratory, Nabeul, Tunisia; Ruđer Bošković Institute: Institut Ruder Boskovic, CROATIA

## Abstract

**Objectives:**

This study aims to assess the performance of the Xpert HPV test for the detection and genotyping of high-risk HPVs (HR-HPV) on FFPE cervical cancer (CC) tissues and to determine the distribution of HR-HPV genotypes in a CC Tunisian series.

**Methods:**

The Xpert HPV test was conducted on purified DNA from 134 FFPE CC tissues. A single-tube multiplex polymerase chain reaction (PCR) served as a comparator assay to evaluate Xpert HPV performance in detecting and genotyping HR-HPVs. Discordant cases were tested using uniplex PCR.

**Results:**

The Xpert HPV test generated valid results in 131/134 (97.8%) samples. Sensitivity, specificity, positive and negative predictive values of Xpert HPV for HR-HPV detection among valid samples were 99.16%, 100%, 100%, and 92.31%, respectively. Agreement between Xpert HPV and multiplex PCR for HPV detection was observed in 131/134 cases (97.8%), with a kappa value of 0.892. Agreement between Xpert HPV and Multiplex PCR for HR-HPV genotyping was 96.6%. Combining assays results and after confirmation with uniplex PCR, HR-HPVs were detected in 119/134 (88.8%) samples and 11 HR-HPV genotypes were detected with a predominance of HPV 16 (71%), followed by HPV 31 (8%) and HPV 18 (5%).

**Conclusion:**

This study demonstrated a high accuracy of the Xpert-HPV test for HR-HPVs detection and genotyping in FFPE CC tissues and revealed a distinct distribution of HR-HPVs in Tunisia.

## Introduction

Cervical cancer (CC) stands as the fourth most diagnosed cancer and the fourth leading cause of cancer-related deaths among women worldwide [1]. Persistent infection with human papillomavirus (HPV) has been identified as the main etiological factor of CC [2]. With over 220 genotypes circulating silently across populations, HPV distribution exhibits a large variability, depending on geographic regions and ethnicities [3]. In Tunisia, CC is the third most common cancer among women, but studies on HPV prevalence and genotype distribution in CC lesions are limited [4–7], highlighting the need for comprehensive data in the Tunisian context.

Studies have shown that HPV-independent CCs exhibit more aggressive clinical features and higher rates of treatment failure than HPV-associated CC [8,9]. It is therefore recommended that HPV status be a standard component of CC pathology reports [10].

Formalin-fixed paraffin-embedded (FFPE) tissues are routinely available for histopathological examination in clinical practice. Although more than 400 HPV tests are currently available, only few are recommended for use in clinical routine and fewer have been validated for FFPE material [11,12]. The Xpert HPV test (Cepheid, Inc. Sunnyvale, CA), is a fully automated test that detects and partially genotypes fourteen high-risk HPVs (HR-HPV) in less than one hour. While its performance has been widely assessed on cervical swabs [13–15], few studies have evaluated its performance on cervical FFPE tissues [16].

The aim of this study was to assess the reliability of the Xpert HPV test for the detection and genotyping of HR-HPVs on FFPE CC tissues and to determine the distribution of HR-HPV genotypes in a CC Tunisian series.

## Methods

### 1. Sample collection and DNA extraction

A series of 134 FFPE CC tissues, corresponding to 16 adenocarcinomas (In situ adenocarcinoma: n = 1; invasive adenocarcinoma: n = 15) and 118 squamous cell carcinomas of the cervix (In situ squamous cell carcinomas: n = 25; invasive squamous cell carcinomas: n = 93) was included (S1A Table). These cases were diagnosed between 2009 and 2024. The median age of patients was 54 years with ages ranging from 25 to 83 years. FFPE tissues were retrieved from the archives of the pathology departments of the Charles Nicolle hospital of Tunis (n = 107) and two pathology laboratories in Nabeul: Taher Maamouri hospital (n = 7) and a private histopathology department (n = 20) in the period between December 2023 and November 2024. In all sites, specimens were processed following standard pathology protocols with fixation in 10% neutral-buffered formalin prior to paraffin embedding. The corresponding hematoxylin-eosin slides were reviewed by a pathologist to select areas containing tumoral tissue, avoiding poorly fixed and necrotic zones. Cervical surgical specimens (n = 97) were manually macro-dissected using a mechanical punch (0.6–0.8 mm diameter) and cervical biopsies (n = 37) were micro-dissected in five sections 5-µm thickness that were recovered in a 1.5 ml sterile labelled tube. To avoid samples cross contamination, blocks were handled with gloves and new disposable microtome blades were used for each case and the bloc holder and the plate of the microtome were disinfected between consecutive cases. DNA extraction was performed with QIAamp® DNA FFPE Tissue (Qiagen). The deparaffinization step was performed using a high-heat pre-treatment in a 150 µl ATL buffer for 15 min at 90°C. After incubation, tubes were briefly centrifuged, the liquid solution was transferred in a sterile tube and 40 µl of proteinase K was added then the total solution was incubated at least for one hour at 65 °C. Subsequent steps were performed according to the manufacturer’s instructions. Quantitative and purity controls of the extracted DNA were assessed by Nanodrop® ND-1000.

### 2. Xpert® HPV test

The Xpert® HPV test (Cepheid, Inc. Sunnyvale, CA) was performed using purified DNA as described in the study of Guerendiain et al. [17]. Twenty-five microliters of nucleic acids extract were diluted with DNAse/RNAse free water to obtain a final volume of 1 ml, was transferred to the sample chamber of the Xpert HPV cartridge. This test is based on a real-time PCR that targets a sequence of 80–150 bp in the E6/E7 region of the viral genome, depending on the genotype [18]. It provides individual genotyping results for HPV 16 (P1) and groups the other HR-HPVs genotypes as follows: P2 (HPV 18 and HPV 45), P3 (HPV 31, HPV 33, HPV 35, HPV 52, and HPV 58), P4 (HPV 51 and HPV 59), and P5 (HPV 39, HPV 56, HPV 66, and HPV 68). A sample adequacy control is included in the cartridge. It detects the presence of a single copy human gene, HMBS to confirm that the sample contains enough human DNA of sufficient quality and free of PCR inhibitors, to perform a qualitative assessment of HPV status. Test results are invalid if amplification of both, the sample adequacy control and HPV DNA is failed.

### 3. Multiplex PCR

An in-house single-tube multiplex PCR, targeting 14 HR-HPV genotypes and an internal control CYP2C8 was used as comparator assay to assess the performance of the Xpert HPV test in HR-HPV detection and genotyping. Triple primed multiplex PCR was employed using three sets of primers for each target: a forward specific primer, a reverse specific primer with a tail on its 5’ end and a universal fluorescently labelled primer (Table 1 and S1 Fig). Universal primers, M13 and M13 (−21), were chosen so that they do not anneal neither to HPV genome nor to the human DNA. Different fluorophores were used to discriminate amplicons with close sizes. A primer mix of the 14 HR-HPV specific primers [19] and CYP2C8 was prepared. It included 0.2 µM of each reverse specific primer and 2 µM of each specific forward and universal primers. PCR was performed with the Multiplex PCR kit (QIAGEN), according to the manufacturer’s instructions. A volume of 25 μl containing 12.5 µl of master mix, 5 µl of the primer mix, 2.5 µl of Q-solution, 2.5 µl of coralLoad dye, 2.5 µl of template DNA (40ng/ µl), was prepared. PCR program was as follows: After a 5 min initial denaturation at 95°C, 40 cycles of amplification were used including a denaturation step at 95 °C for 30 s, an annealing step at 60°C for 1 min and an elongation step at 72 °C for 30 s. Then, a final extension step at 68°C for 15 min was performed.

**Table 1:** Multiplex PCR primer sequences, targets and product lengths.

Primer name	Sequence (5’-3’)	Target	Product’s in silico predicted size (nt)
HPV 16	F: GTTGCAGATCATCAAGAACACG R:TACGCATCCCAGTTTGAGACGCATCCTCCTCCTCTGAGCTG	E6-E7 Region	152
HPV 18	F: AGTGCCATTCGTGCTGCAAC R:TACGCATCCCAGTTTGAGACGATGTTGCCTTAGGTCCATGCAT		119
HPV 31	F: GTGGACAGGACGTTGCATAGYA R:TACGCATCCCAGTTTGAGACGGGTCAGTTGCCTCAGGTTGCA		145
HPV 33	F: AATATTTCGGGTCGTTGGGC R:TGTAAAACGACGGCCAGTAACGTTGGCTTGTGTCCTCTCA		127
HPV 35	F: GGTGGACAGGTCGGTGTATGTC R:TACGCATCCCAGTTTGAGACGGTTGCCTCGGGTTCCAAATC		141
HPV 39	F: ACAGTGTCGACGGTGCTGGA R:TACGCATCCCAGTTTGAGACGGCTTTGGTCCACGCATATCTGA		115
HPV 45	F: GGACAGTACCGAGGGCAGTGTA R:TACGCATCCCAGTTTGAGACGCCGGGGTCCATGCATACTTAT		128
HPV 51	F: AATGCGCTAATTGCTGGC R:TGTAAAACGACGGCCAGTGTGGTGTTAAATGCAATACTACATCT		130
HPV 52	F: GTTGGACAGGGCGCTGTTC R:TACGCATCCCAGTTTGAGACGCCTCCTCATCTGAGCTGTCACC		187
HPV 56	F: TGGTTGGACCGGGTCATGT R:TGTAAAACGACGGCCAGTCGTCTTGCAGCGTTGGTACTTT		121
HPV 58	F: AGGGCGCTGTGCAGTGTGT R:TACGCATCCCAGTTTGAGACGCATCCTCGTCTGAGCTGTCACA		193
HPV 59	F: ACAGTGTCGTGGGTGTCGGA R:TGTAAAACGACGGCCAGTTGCTCGTAGCACACAAGGTCAA		187
HPV 66	F: ACCGGGTCATGTTTGCAGTGT R:TGTAAAACGACGGCCAGTCGTTTGCGGTGCAAGTTCTAAT		140
HPV 68	F: ACAGTGTCGSCACTGCTGGA R:TGTAAAACGACGGCCAGTGGGCTTTGGTCCATGCATAGT		115
CYP2C8	F: GAACACCAAGCATCACTGGA R:TGTAAAACGACGGCCAGTGAAATCAAAATACTGATCTGTTGC	Human gene	128
M13	FAM-TACGCATCCCAGTTTGAGACG-3’	–	–
M13 (−21)	HEX-TGTAAAACGACGGCCAGT-3’	–	–

PCR products were then analysed using capillary electrophoresis. In each plate well, 1 μl of PCR product was added to a mixture of 10 μl formamide and 0.3 μl GeneScan™- 600 size standard (applied Biosystem). Samples were denatured 5 min at 95°C, immediately placed on ice for 5 min and loaded on the Applied Biosystems® 3500 genetic analyser (Faculty of Medicine of Tunis). The raw FSA data files have been uploaded to GeneMarker® V2.2 and analysed following the amplified fragment length polymorphism (AFLP) method. Peaks were retained if their relative fluorescence units were ≥500. HPV genotypes were identified basing on sizes and fluorescence types of the corresponding peaks.

### 4. Discrepant analysis

HPV genotyping was considered discordant if genotypes determined by multiplex PCR were not included in the detected Xpert HPV genotypes groups. In cases of discordance, uniplex PCR with type-specific primers was performed. A volume of 25 μl containing 12.5 µl of master mix, 5 µl of the primer mix, 2.5 µl of Q-solution, 2.5 µl of coralLoad dye and 2.5 µl of template DNA (40ng/ µl), was prepared. PCR program was as follows: After a 5 min initial denaturation at 95°C, 35 cycles of amplification were used including a denaturation step at 95 °C for 30 s, an annealing step at 60°C for 30s and an elongation step at 72 °C for 30 s. PCR products were analysed by capillary electrophoresis as described above.

### 5. Statistical analysis

Cohen’s kappa coefficient with 95% confidence intervals was calculated to assess the agreement between the Xpert HPV test and multiplex PCR for HR-HPV detection regardless of specific HR-HPV genotypes. The obtained kappa values were interpreted as follows: κ < 0.00 indicating poor agreement, κ = 0.00–0.20 indicating slight agreement, κ = 0.21–0.40 indicating fair agreement, κ = 0.41–0.60 indicating moderate agreement, κ = 0.61–0.80 indicating substantial agreement, and κ = 0.81–1.00 indicating almost perfect agreement. To assess whether the distribution of test outcomes (positive, negative, and invalid) differs significantly between the two methods, the Stuart-Maxwell test was applied. Invalid Xpert HPV test cases were discarded to determine the sensitivity, specificity, positive and negative predictive values of the Xpert HPV test with reference to multiplex PCR for HPV detection [20]. The reliability of Xpert HPV for HR-HPV genotyping was assessed with reference to multiplex PCR and uniplex PCR for discordant cases. The distribution of HR-HPV genotypes was reported descriptively.

### 6. Ethical consideration

The study was approved by the local Ethics Committee of Charles Nicolle Hospital in Tunis and conducted in accordance with good clinical practice guidelines. Informed consent could not be obtained as the data were retrospectively collected. No direct patient contact or intervention occurred, and all identifiable information was removed prior to access and use for research purposes to ensure participant confidentiality.

## Results

### 1. Assays validity and DNA yield

Xpert HPV generated valid results for 131/134 (97.8%) FFPE specimens. Nucleic acid yields ranged from 25 to 2225 ng/µl. Three samples (2.2%) produced invalid results despite having acceptable DNA quantity and A260/A280 ratios (sample 1: 202.3 ng/µl, A260/A280 = 1.94; sample 2: 43.2 ng/µl, A260/A280 = 2.06; sample 3: 909.5 ng/µl, A260/A280 = 1.9), and remained invalid after retesting with increased DNA amount. One hundred thirty three (99.3%) samples were valid using multiplex PCR. Among the three Xpert HPV invalid samples, two were valid but HPV negative using multiplex PCR and the third was invalid with both assays.

### 2. Performance of Xpert HPV for HR-HPV detection

Using Xpert HPV, 118/134 (88.1%) were HR-HPV positive and 13/134 (9.7%) samples were HR-HPV negative. Using multiplex PCR, 119/134 (88.8%) samples were HR-HPV positive and 14/134 (10.4%) were HR-HPV negative. Concordant results were observed in 131/134 samples (97.8%) with a kappa value of 0.892 (95% confidence interval: 0.779–1.000), indicating an almost perfect agreement (Table 2). The Stuart-Maxwell test revealed no significant asymmetry in the discordant results (χ² = 3.0, df = 2, p = 0.22), suggesting that the discrepancies were random and not systematic.

**Table 2:** HR-HPV detection by Xpert HPV vs multiplex PCR.

		Multiplex PCR		
		Positive	Negative	Invalid
**Xpert HPV**	Positive	118	0	0
	Negative	1	12	0
	Invalid	0	2	1

One case was HR-HPV positive by multiplex PCR but negative by Xpert HPV, indicating an Xpert HPV false-negative result. No false positive cases were detected (S1B Table). The Xpert HPV test demonstrated a sensitivity of 99.16%, a specificity of 100%, a positive predictive value of 100%, and a negative predictive value of 92.31% for detecting HR-HPV.

### 3. Performance of the Xpert HPV for HR-HPV genotyping and HR-HPV genotypes distribution

**Fig 1:**
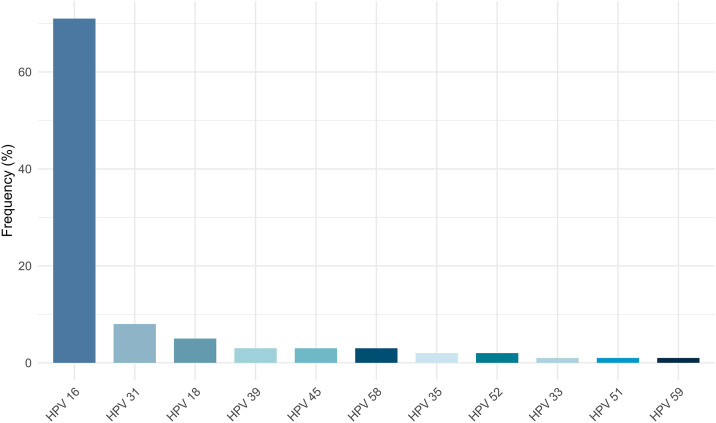
HR-HPV genotypes distribution in CC lesions.

Using Xpert HPV, HPV16 was detected in 94/118 samples (74.2%), P3 (HPV 31, 33, 35, 52, and 58) in 19/118 samples (14.8%), P2 (HPV 18 and 45) in 11/118 samples (8.6%), P4 (HPV 51 and 59) in 2/118 samples (1.6%), and P5 (HPV 39, 56, 66, and 68) in 1/118 sample (0.8%). Genotyping with multiplex PCR revealed the presence of 11 HR-HPV: HPV 16 was identified in 94/119 HR-HPV positive samples (71%), followed respectively by HPV 31, detected in 11/119 samples (8%), and HPV 18, detected in 7 samples (5%), HPV 56, HPV 66, and HPV 68 were not detected in the present study (Fig 1). HPV 16, 18, 31,33,45,52 and 58 accounted together for 93.9%. Co-infection with two HPV genotypes was identified in 11 cases. HPV 16 was present in nine of these cases, each paired with one of the following genotypes: HPV 31, 35, 39, 45, 51, 52, or 58. The remaining two double infections involved HPV 59 and HPV 31, and HPV 31 with HPV 35. Additionally, one case exhibited a triple infection with HPV 31, HPV 16, and HPV 39. Notably, HPV 35 and HPV 51 were detected only in cases of multiple infections.

Agreement between Xpert HPV genotypes groups and multiplex PCR genotypes was observed in 96.6% of samples, with perfect agreement for HPV16, P2, and P4. Discordant results were observed in four cases: one was a false negative case and the three others were multiple infection cases in which the Xpert HPV test identified only one genotypes group (table 3).

**Table 3:** Genotype discrepancies between Xpert HPV and multiplex PCR.

Sample	Xpert HPV test	Multiplex PCR
CC15	P3*, HPV 16	HPV 16, HPV 31, and **HPV 39**
CC27	Negative	**HPV 39**
CC41	P4 **	**HPV 31** and HPV 59
CC72	HPV 16	HPV 16 and **HPV 39**

*: Xpert HPV P3: HPV 31, HPV 33, HPV 35, HPV 52, and HPV 58; **: Xpert HPV P4: HPV 51 and HPV 59

## Discussion

This study demonstrated the accuracy of Xpert HPV for HR-HPV detection and genotyping in FFPE CC tissues. This test exhibited almost perfect agreement with multiplex PCR for HR-HPV detection and high agreement for HR-HPV genotyping. To the best of our knowledge, there have been no published studies on the performance of the Xpert HPV test on FFPE CC tissues. Only one study has evaluated the performance of this test on FFPE precancerous cervical lesions and two other studies have examined its performance on FFPE oropharyngeal carcinomas tissues [16–18].

The determination HPV status in CC lesions is a determinant of CC prognosis and treatment response [21–23]. HPV genotyping in CC may also have implications in patient management: CC patients infected with HPV 18 were proven to have worse survival compared with CC patients with HPV 16, HPV31 and HPV58 infection [24–26]. However, the theranostic significance of the other HPV genotypes is not well known [26].

Various methods are available to determine HPV status. Immunohistochemical expression of p16INK4a is widely used in FFPE tissues. However, this biomarker lacks specificity and can exhibit overexpression in normal tissues and in a subset of HPV-negative tumors [27–29]. HPV DNA detection provides direct evidence of viral infection. Numerous molecular assays have been developed for HPV detection and validated on cervical swabs [11,12], but require validation on FFPE tissues for routine clinical use. Formalin fixation induces significant DNA damage, including cross-linking and fragmentation. Therefore, PCR amplicons should not exceed 300 bp [30,31], which aligns with the specifications of the Xpert HPV test targeting 75–150 bp of specific HR-HPV sequences, and the multiplex PCR used as a comparator assay in this study.

Xpert HPV invalid results rate in this study was 2.2%. This rate is lower than those reported by Guerendiain et al. (9.1%) [17] and Virtanen et al (16.7%) [16]. Invalid tests rate in the present study is in line with those observed in the screening population (3%) [32]. Although their relatively low rate, invalid tests may have several drawbacks such as repeated testing, diagnostic delay, additional costs and missed positive cases [33]. These issues underscore the need to adjust preanalytical procedures particularly in FFPE tissues.

The concordance between Xpert HPV and multiplex PCR for HR-HPV detection was high. When comparing HPV assays, discordant cases are not uncommon in CC screening: the Danish split-sample Horizon study, comparing four HPV assays in women undergoing primary CC screening, showed that only 29% of all positive tests results were concordant on all four compared assays. This is mainly due to differences in assays technologies, targeted sequences, amplicon sizes, cross reactivity and samples pre-treatment [34]. However, consistent with our findings, when HPV tests are compared in high-grade cervical lesions or CC, they generally exhibit very low discordance, ensuring that severe cervical lesions are not missed [35].

In this study, four discordant cases in HPV genotyping were observed. Three of these four cases were multiple infections. Commercially available HPV tests may fail to reliably detect and differentiate HR-HPV multiple infections. This limitation may be due to technical factors, such as competitive inhibition during amplification and reduced sensitivity for less dominant genotypes in a multiple infection [36,37]. HPV39 was found in three out of four discrepant cases. This discrepancy may be related to intratypic HPV39 variants prevalent in Tunisia, which suboptimally detected by Xpert HPV. However, this hypothesis cannot be validated in the present study, as HPV sequencing was not performed. Although assays are designed to target conserved regions, nucleotide polymorphisms within these loci may lead to decreased primer and probe binding affinity. Jiang et al, 2009, demonstrated that HPV16 intratypic variation can critically impair assay performance through primer-target mismatches, leading to inaccurate viral load and integration metrics [38].

In the present series, 11 HR-HPV types were detected, HPV 16 was the most prevalent genotype (71%) which aligns with Tunisian and global data identifying HPV 16 as the most attributable genotype to CC [4,5,7,39]. HPV 18 identified second most prevalent genotype worldwide (10%), was less frequent in our cohort (5%). HPV31, the second most prevalent genotype in this study (8%), accounts for 4% of all CC cases worldwide [39]. Together, HPV 16, 18, 31,33,45,52 and 58, the nonavalent vaccine targeted genotypes, accounted for 93.9% of cases in this study; which is close to the global attribution of these genotypes to CC (94.7%) [39]. While the individual prevalence of other HR-HPV (HPV 35, 39, 51, and 59) is low, they collectively account for 6.1% of CC cases in this study. HPV 35, detected in 2% of CC in this study and present only in multiple infection cases, holds particular importance in sub-Saharan Africa, where it is more commonly found in CC cases than in other regions [40]. These genotypes contribute to the residual risk of CC and they show a high regional variability. This residual risk highlights the need for ongoing surveillance and consideration of expanded vaccine formulations to address these less common but oncogenic HPV types effectively.

In conclusion, this study highlights the reliability of the Xpert HPV test for HR-HPV detection and genotyping in CC FFPE tissues, making it a good candidate for use in clinical sittings. Additionally, it shows a distinct HR-HPV genotypes distribution and provides valuable insights for health policymakers to implement comprehensive and effective prevention strategies.

## Supporting information

S1 Table S1A. Study cohort and HPV testing results by multiplex PCR and Xpert HPV, S1B. Diagnostic performance of the Xpert HPV test on formalin-fixed paraffin-embedded tissues of the cervix with reference to multiplex PCR.
(DOCX)

S1 Fig Schematic representation of the triple-primed multiplex PCR strategy. (PDF)

## References

[1] Sung H, Ferlay J, Siegel RL, Laversanne M, Soerjomataram I, Jemal A, et al. Global Cancer Statistics 2020: GLOBOCAN Estimates of Incidence and Mortality Worldwide for 36 Cancers in 185 Countries. CA Cancer J Clin. 2021;71(3):209–49. doi: 10.3322/caac.21660 33538338

[2] Walboomers JM, Jacobs MV, Manos MM, Bosch FX, Kummer JA, Shah KV, et al. Human papillomavirus is a necessary cause of invasive cervical cancer worldwide. J Pathol. 1999;189(1):12–9. doi: 10.1002/(SICI)1096-9896(199909)189:1<12::AID-PATH431>3.0.CO;2-F 10451482

[3] Tang S, Liao Y, Hu Y, Shen H, Wan Y, Wu Y. HPV Prevalence and Genotype Distribution Among Women From Hengyang District of Hunan Province, China. Front Public Health. 2021;9:710209. 34805062 10.3389/fpubh.2021.710209 PMC8602211

[4] KrennHrubec K, Mrad K, Sriha B, Ben Ayed F, Bottalico DM, Ostolaza J, et al. HPV Types and Variants Among Cervical Cancer Tumors in Three Regions of Tunisia. J Med Virol.2011;83(4):651‑7. 21328380 10.1002/jmv.22011 PMC4291031

[5] Missaoui N, Hmissa S, Trabelsi A, Tahar Yacoubi M, Nouira A, Frappart L, et al. Prevalence of HPV infection in precancerous and cancerous lesions of the uterine cervix in Tunisia. Ann Biol Clin (Paris). 2010;68(3):297–303. doi: 10.1684/abc.2010.0431 20478773

[6] Ennaifer E, Salhi F, Laassili T, Fehri E, Ben Alaya N, Guizani I, et al. Type-Specific Human Papillomavirus Distribution in Invasive Squamous Cervical Carcinomas in Tunisia and Vaccine Impact. Asian Pac J Cancer Prev. 2015;16(15):6769–72. doi: 10.7314/apjcp.2015.16.15.6769 26434909

[7] Bel Haj Rhouma R, Ardhaoui M, El Fehri E, Marzougui A, Laassili T, Guizani I, et al. Distribution of human papillomavirus in precancerous and cancerous cervical neoplasia in Tunisian women. Infect Agent Cancer. 2021;16(1):52. doi: 10.1186/s13027-021-00392-1 34271960 PMC8283945

[8] Mkrtchian L, Zamulaeva I, Krikunova L, Kiseleva V, Matchuk O, Liubina L, et al. HPV Status and Individual Characteristics of Human Papillomavirus Infection as Predictors for Clinical Outcome of Locally Advanced Cervical Cancer. J Pers Med. 2021;11(6):479. doi: 10.3390/jpm11060479 34071821 PMC8227948

[9] Arezzo F, Cormio G, Loizzi V, Cazzato G, Cataldo V, Lombardi C, et al. HPV-Negative Cervical Cancer: A Narrative Review. Diagnostics (Basel). 2021;11(6):952. doi: 10.3390/diagnostics11060952 34073478 PMC8229781

[10] World Health Organization. Classification of tumours: female genital tumours [Internet]. 5th ed. Lyon, France: International Agency for Research on Cancer; 2020 [cited 2024 May 27]. Available from: https://publications.iarc.fr/Book-And-Report-Series/Who-Classification-Of-Tumours/Female-Genital-Tumours-2020

[11] Poljak M, Oštrbenk Valenčak A, Gimpelj Domjanič G, Xu L, Arbyn M. Commercially available molecular tests for human papillomaviruses: a global overview. Clin Microbiol Infect. 2020;26(9):1144–50. doi: 10.1016/j.cmi.2020.03.033 32247892

[12] Arbyn M, Simon M, Peeters E, Xu L, Meijer CJLM, Berkhof J, et al. 2020 list of human papillomavirus assays suitable for primary cervical cancer screening. Clin Microbiol Infect. 2021;27(8):1083–95. doi: 10.1016/j.cmi.2021.04.031 33975008

[13] Akbari A, Vanden Broeck D, Benoy I, Padalko E, Bogers J, Arbyn M. Validation of intra- and inter-laboratory reproducibility of the Xpert HPV assay according to the international guidelines for cervical cancer screening. Virol J. 2018;15(1):166. doi: 10.1186/s12985-018-1076-6 30373616 PMC6206920

[14] Rabaan AA, Taylor DR, Dawamneh MF, Al-Tawfiq JA. Comparison of Xpert® HPV and Hybrid Capture® 2 DNA Test™ for detection of high-risk HPV infection in cervical atypical squamous cells of undetermined significance. J Infect Public Health. 2017;10(2):219–23. doi: 10.1016/j.jiph.2016.04.017 27343005

[15] Latsuzbaia A, Vanden Broeck D, Van Keer S, Weyers S, Donders G, Doyen J, et al. Comparison of the Clinical Accuracy of Xpert HPV Assay on Vaginal Self-Samples and Cervical Clinician-Taken Samples within the VALHUDES Framework. J Mol Diagn. 2023;25(9):702–8. doi: 10.1016/j.jmoldx.2023.06.004 37354994

[16] Virtanen E, Laurila P, Hagström J, Nieminen P, Auvinen E. Testing for high-risk HPV in cervical and tonsillar paraffin-embedded tissue using a cartridge-based assay. APMIS. 2017;125(10):910–5. doi: 10.1111/apm.12727 28736821

[17] Guerendiain D, Moore C, Wells L, Conn B, Cuschieri K. Formalin fixed paraffin embedded (FFPE) material is amenable to HPV detection by the Xpert(®) HPV assay. J Clin Virol. 2016;77:55–9. doi: 10.1016/j.jcv.2016.02.007 26896874

[18] Donà MG, Rollo F, Pichi B, Spriano G, Pellini R, Covello R, et al. Evaluation of the Xpert® HPV assay in the detection of Human Papillomavirus in formalin-fixed paraffin-embedded oropharyngeal carcinomas. Oral Oncol. 2017;72:117–22. doi: 10.1016/j.oraloncology.2017.07.016 28797447

[19] Dictor M, Warenholt J. Single-tube multiplex PCR using type-specific E6/E7 primers and capillary electrophoresis genotypes 21 human papillomaviruses in neoplasia. Infect Agent Cancer. 2011;6(1):1. doi: 10.1186/1750-9378-6-1 21241508 PMC3035480

[20] Shinkins B, Thompson M, Mallett S, Perera R. Diagnostic accuracy studies: how to report and analyse inconclusive test results. BMJ. 2013;346:f2778. doi: 10.1136/bmj.f2778 23682043

[21] Fernandes A, Viveros-Carreño D, Hoegl J, Ávila M, Pareja R. Human papillomavirus-independent cervical cancer. Int J Gynecol Cancer. 2022;32(1):1–7. doi: 10.1136/ijgc-2021-003014 34725203

[22] Lei J, Arroyo-Mühr LS, Lagheden C, Eklund C, Nordqvist Kleppe S, Elfström M, et al. Human Papillomavirus Infection Determines Prognosis in Cervical Cancer. J Clin Oncol. 2022;40(14):1522–8. doi: 10.1200/JCO.21.01930 35077203

[23] Li P, Tan Y, Zhu L-X, Zhou L-N, Zeng P, Liu Q, et al. Prognostic value of HPV DNA status in cervical cancer before treatment: a systematic review and meta-analysis. Oncotarget. 2017;8(39):66352–9. doi: 10.18632/oncotarget.18558 29029517 PMC5630417

[24] Huang Y, Zou D, Guo M, He M, He H, Li X, et al. HPV and radiosensitivity of cervical cancer: a narrative review. Ann Transl Med. 2022;10(24):1405. doi: 10.21037/atm-22-5930 36660629 PMC9843372

[25] Xu Y, Qiu Y, Yuan S, Wang H. Prognostic implication of human papillomavirus types in cervical cancer patients: a systematic review and meta-analysis. Infect Agent Cancer. 2020;15(1):66. doi: 10.1186/s13027-020-00332-5 33292343 PMC7648311

[26] Onuki M, Matsumoto K, Tenjimbayashi Y, Tasaka N, Akiyama A, Sakurai M, et al. Human papillomavirus genotype and prognosis of cervical cancer: Favorable survival of patients with HPV16-positive tumors. Papillomavirus Res. 2018;6:41–5. doi: 10.1016/j.pvr.2018.10.005 30347290 PMC6218653

[27] Cantley RL, Gabrielli E, Montebelli F, Cimbaluk D, Gattuso P, Petruzzelli G. Ancillary studies in determining human papillomavirus status of squamous cell carcinoma of the oropharynx: a review. Patholog Res Int. 2011;2011:138469. doi: 10.4061/2011/138469 21772959 PMC3137958

[28] Nicolás I, Marimon L, Barnadas E, Saco A, Rodríguez-Carunchio L, Fusté P, et al. HPV-negative tumors of the uterine cervix. Mod Pathol. 2019;32(8):1189–96. doi: 10.1038/s41379-019-0249-1 30911077

[29] Pereira Pinto P, Zanine RM. Diagnostic value of p16 and Ki-67 expression in cervical glandular intraepithelial disease: A review. Ann Diagn Pathol. 2023;62:152054. doi: 10.1016/j.anndiagpath.2022.152054 36396551

[30] Dietrich D, Uhl B, Sailer V, Holmes EE, Jung M, Meller S, et al. Improved PCR performance using template DNA from formalin-fixed and paraffin-embedded tissues by overcoming PCR inhibition. PLoS One. 2013;8(10):e77771. doi: 10.1371/journal.pone.0077771 24155973 PMC3796491

[31] van Deventer BS, du Toit-Prinsloo L, van Niekerk C. Practical tips to using formalin-fixed paraffin-embedded tissue archives for molecular diagnostics in a South African setting. Afr J Lab Med. 2022;11(1):1587. doi: 10.4102/ajlm.v11i1.1587 35811747 PMC9257738

[32] Cuzick J, Cuschieri K, Denton K, Hopkins M, Thorat MA, Wright C, et al. Performance of the Xpert HPV assay in women attending for cervical screening. Papillomavirus Research. 2015;1:32–7. doi: 10.1016/j.pvr.2015.05.002

[33] Cuschieri K, Wilson A, Palmer T, Stanczuk G, Bhatia R, Ejegod D, et al. The challenges of defining sample adequacy in an era of HPV based cervical screening. J Clin Virol. 2021;137:104756. doi: 10.1016/j.jcv.2021.104756 33662921

[34] de Thurah L, Bonde J, Lam JUH, Rebolj M. Concordant testing results between various human papillomavirus assays in primary cervical cancer screening: systematic review. Clin Microbiol Infect. 2018;24(1):29–36. doi: 10.1016/j.cmi.2017.05.020 28559000

[35] Rebolj M, Bonde J, Preisler S, Ejegod D, Rygaard C, Lynge E. Human Papillomavirus Assays and Cytology in Primary Cervical Screening of Women Aged 30 Years and Above. PLoS One. 2016;11(1):e0147326. doi: 10.1371/journal.pone.0147326 26789267 PMC4720421

[36] Shen-Gunther J, Wang Y, Lai Z, Poage GM, Perez L, Huang THM. Deep sequencing of HPV E6/E7 genes reveals loss of genotypic diversity and gain of clonal dominance in high-grade intraepithelial lesions of the cervix. BMC Genomics. 2017;18(1):231. doi: 10.1186/s12864-017-3612-y 28288568 PMC5348809

[37] Pesic A, Krings A, Hempel M, Preyer R, Chatzistamatiou K, Agorastos T, et al. CIN2+ detection of the HPV DNA Array genotyping assay in comparison with the Cobas 4800 HPV test and cytology. Virol J. 2019;16(1):92. doi: 10.1186/s12985-019-1197-6 31337408 PMC6651913

[38] Jiang M, Baseman JG, Koutsky LA, Feng Q, Mao C, Kiviat NB, et al. Sequence variation of human papillomavirus type 16 and measurement of viral integration by quantitative PCR. J Clin Microbiol. 2009;47(3):521–6. doi: 10.1128/JCM.02115-08 19116350 PMC2650947

[39] de Sanjose S, Quint WG, Alemany L, Geraets DT, Klaustermeier JE, Lloveras B, et al. Human papillomavirus genotype attribution in invasive cervical cancer: a retrospective cross-sectional worldwide study. Lancet Oncol. 2010;11(11):1048–56. doi: 10.1016/S1470-2045(10)70230-8 20952254

[40] Wei F, Georges D, Man I, Baussano I, Clifford GM. Causal attribution of human papillomavirus genotypes to invasive cervical cancer worldwide: a systematic analysis of the global literature. Lancet. 2024;404(10451):435–44. doi: 10.1016/S0140-6736(24)01097-3 39097395

