# Supplementary material for: HPV testing in cervical cancer formalin-fixed paraffin embedded tissues: Reliability of the Xpert HPV test and high-risk HPV genotypes distribution in Tunisia
Source: PLoS One. 2025 Oct 24;20(10):e0333600. doi: 10.1371/journal.pone.0333600 (PMC12551861; doi:10.1371/journal.pone.0333600)
Supplement: S1 Table — S1A. Study cohort and HPV testing results by multiplex PCR and Xpert HPV, S1B. Diagnostic performance of the Xpert HPV test on formalin-fixed paraffin-embedded tissues of the cervix with reference to multiplex PCR. (DOCX) [file pone.0333600.s001.docx]

**S1A Table. Study cohort and HPV testing results by multiplex PCR and Xpert HPV**

| Patient | Storage duration of FFPE blocks (years) | Histological type | Xpert HPV | Multiplex PCR |
| --- | --- | --- | --- | --- |
| CC1 | 6 | Squamous cell carcinoma | P1 | HPV 16 |
| CC2 | 7 | Squamous cell carcinoma | P1 | HPV 16 |
| CC3 | 6 | Squamous cell carcinoma | P1 | HPV 16 |
| CC4 | 14 | Adenocarcinoma | P1 | HPV 16 |
| CC5 | 6 | Squamous cell carcinoma | P1 | HPV 16 |
| CC6 | 5 | Squamous cell carcinoma | P1 | HPV 16 |
| CC7 | 6 | Squamous cell carcinoma | P1 | HPV 16 |
| CC8 | 7 | Squamous cell carcinoma | P1 | HPV 16 |
| CC9 | 7 | Squamous cell carcinoma | P1 | HPV 16 |
| CC10 | 11 | Squamous cell carcinoma | P1 p4 | HPV 16 HPV 51 |
| CC11 | 10 | Squamous cell carcinoma | P1 p3 | HPV 16 HPV 58 |
| CC12 | 15 | Squamous cell carcinoma | P1 p2 | HPV 16 HPV 45 |
| CC13 | 12 | Squamous cell carcinoma | P1 | HPV 16 |
| CC14 | 5 | Squamous cell carcinoma | P1 | HPV 16 |
| CC15 | 6 | Squamous cell carcinoma | P1 p3 | HPV 16 HPV 31 **HPV 39** |
| CC16 | 3 | Squamous cell carcinoma | P1 | HPV 16 |
| CC17 | 3 | Squamous cell carcinoma | P1 | HPV 16 |
| CC18 | 2 | Squamous cell carcinoma | P1 | HPV 16 |
| CC19 | 12 | Squamous cell carcinoma | p2 | HPV 45 |
| CC20 | 14 | Squamous cell carcinoma | p3 | HPV 52 |
| CC21 | 6 | Squamous cell carcinoma | P1 | HPV 16 |
| CC22 | 3 | Squamous cell carcinoma | P1 | HPV 16 |
| CC23 | 14 | Squamous cell carcinoma | P1 | HPV 16 |
| CC24 | 2 | Squamous cell carcinoma | p3 | HPV 31 |
| CC25 | 11 | Squamous cell carcinoma | p3 | HPV 58 |
| CC26 | 3 | Squamous cell carcinoma | P1 | HPV 16 |
| CC27 | 3 | Squamous cell carcinoma | negative | **HPV 39** |
| CC28 | 3 | Squamous cell carcinoma | P1 p5 | HPV 16 HPV 39 |
| CC29 | 6 | Adenocarcinoma | P1 | HPV 16 |
| CC30 | 8 | Squamous cell carcinoma | P1 | HPV 16 |
| CC31 | 6 | Squamous cell carcinoma | p3 | HPV 31 |
| CC32 | 2 | Squamous cell carcinoma | P1 | HPV 16 |
| CC33 | 10 | Squamous cell carcinoma | P1 | HPV 16 |
| CC34 | 3 | Squamous cell carcinoma | P1 | HPV 16 |
| CC35 | 7 | Squamous cell carcinoma | P1 | HPV 16 |
| CC36 | 3 | Squamous cell carcinoma | P1 | HPV 16 |
| CC37 | 4 | Squamous cell carcinoma | invalid | Negative |
| CC38 | 6 | Squamous cell carcinoma | p2 | HPV 45 |
| CC39 | 14 | Squamous cell carcinoma | P1 | HPV 16 |
| CC40 | 8 | Squamous cell carcinoma | P1 | HPV 16 |
| CC41 | 5 | Squamous cell carcinoma | p4 | **HPV 31** HPV 59 |
| CC42 | 2 | Squamous cell carcinoma | P1 | HPV 16 |
| CC43 | 7 | Squamous cell carcinoma | p3 | HPV 31 |
| CC44 | 9 | Squamous cell carcinoma | P1 | HPV 16 |
| CC45 | 3 | Squamous cell carcinoma | P1 | HPV 16 |
| CC46 | 9 | Squamous cell carcinoma | P1 p3 | HPV 16 HPV 58 |
| CC47 | 7 | Squamous cell carcinoma | P1 | HPV 16 |
| CC48 | 2 | Squamous cell carcinoma | P1 | HPV 16 |
| CC49 | 3 | Squamous cell carcinoma | P1 | HPV 16 |
| CC50 | 7 | Squamous cell carcinoma | negative | negative |
| CC51 | 12 | Squamous cell carcinoma | p3 | HPV 31 |
| CC52 | 8 | Squamous cell carcinoma | P1 | HPV 16 |
| CC53 | 7 | Squamous cell carcinoma | P1 | HPV 16 |
| CC54 | 8 | Squamous cell carcinoma | invalid | negative |
| CC55 | 7 | Squamous cell carcinoma | P1 | HPV 16 |
| CC56 | 7 | Squamous cell carcinoma | P1 | HPV 16 |
| CC57 | 3 | Squamous cell carcinoma | P1 | HPV 16 |
| CC58 | 7 | Squamous cell carcinoma | P1 | HPV 16 |
| CC59 | 7 | Squamous cell carcinoma | P1 | HPV 16 |
| CC60 | 4 | Squamous cell carcinoma | P1 p3 | HPV 16 HPV 35 |
| CC61 | 8 | Squamous cell carcinoma | P1 p3 | HPV 16 HPV 52 |
| CC62 | 8 | Squamous cell carcinoma | P1 | HPV 16 |
| CC63 | 3 | Squamous cell carcinoma | P1 | HPV 16 |
| CC64 | 11 | Squamous cell carcinoma | P1 | HPV 16 |
| CC65 | 7 | Adenocarcinoma | P1 | HPV 16 |
| CC66 | 2 | Squamous cell carcinoma | p2 | HPV 45 |
| CC67 | 8 | Squamous cell carcinoma | P1 | HPV 16 |
| CC68 | 7 | Adenocarcinoma | negative | negative |
| CC69 | 3 | Squamous cell carcinoma | P1 | HPV 16 |
| CC70 | 8 | Adenocarcinoma | P1 | HPV 16 |
| CC71 | 8 | Adenocarcinoma | P1 | HPV 16 |
| CC72 | 3 | Squamous cell carcinoma | P1 | HPV 16 **HPV 39** |
| CC73 | 3 | Squamous cell carcinoma | P1 | HPV 16 |
| CC74 | 4 | Adenocarcinoma | P1 | HPV 16 |
| CC75 | 5 | Squamous cell carcinoma | P1 | HPV 16 |
| CC76 | 3 | Squamous cell carcinoma | P1 | HPV 16 |
| CC77 | 10 | Squamous cell carcinoma | negative | negative |
| CC78 | 2 | Squamous cell carcinoma | P1 | HPV 16 |
| CC79 | 2 | Adenocarcinoma | negative | negative |
| CC80 | 2 | Adenocarcinoma | P1 | HPV 16 |
| CC81 | 2 | Squamous cell carcinoma | P1 | HPV 16 |
| CC82 | 2 | Squamous cell carcinoma | negative | negative |
| CC83 | 2 | Squamous cell carcinoma | P1 | HPV 16 |
| CC84 | 1 | Squamous cell carcinoma | P1 | HPV 16 |
| CC85 | 1 | Squamous cell carcinoma | p2 | HPV 18 |
| CC86 | 1 | Squamous cell carcinoma | P1 | HPV 16 |
| CC87 | 1 | Squamous cell carcinoma | P1 | HPV 16 |
| CC88 | 4 | Adenocarcinoma | negative | negative |
| CC89 | 1 | Squamous cell carcinoma | p2 | HPV 18 |
| CC90 | 2 | Squamous cell carcinoma | P1 | HPV 16 |
| CC91 | 1 | Squamous cell carcinoma | negative | negative |
| CC92 | 1 | Squamous cell carcinoma | P1 | HPV 16 |
| CC93 | 1 | Squamous cell carcinoma | P1 | HPV 16 |
| CC94 | 1 | Adenocarcinoma | P1 | HPV 16 |
| CC95 | 1 | Squamous cell carcinoma | P1 | HPV 16 |
| CC96 | 15 | Squamous cell carcinoma | Invalid | Invalid |
| CC97 | 13 | Squamous cell carcinoma | p3 | HPV 52 |
| CC98 | 9 | Squamous cell carcinoma | P1 p3 | HPV 16 HPV 31 |
| CC99 | 13 | Squamous cell carcinoma | P1 | HPV 16 |
| CC100 | 9 | Squamous cell carcinoma | p3 | HPV 31 |
| CC101 | 13 | Squamous cell carcinoma | p2 | HPV 18 |
| CC102 | 12 | Squamous cell carcinoma | P1 | HPV 16 |
| CC103 | 2 | Adenocarcinoma | p2 | HPV 18 |
| CC104 | 12 | Squamous cell carcinoma | P1 | HPV 16 |
| CC105 | 14 | Adenocarcinoma | negative | negative |
| CC106 | 2 | Adenocarcinoma | P1 | HPV 16 |
| CC107 | 10 | Adenocarcinoma | P1 | HPV 16 |
| CC108 | 1 | Squamous cell carcinoma | p3 | HPV 33 |
| CC109 | 1 | Squamous cell carcinoma | p3 | HPV 58 |
| CC110 | 1 | Squamous cell carcinoma | P1 | HPV 16 |
| CC111 | 1 | Squamous cell carcinoma | P1 | HPV 16 |
| CC112 | 1 | Squamous cell carcinoma | P1 | HPV 16 |
| CC113 | 1 | Squamous cell carcinoma | negative | negative |
| CC114 | 1 | Squamous cell carcinoma | p3 | HPV 31 |
| CC115 | 1 | Squamous cell carcinoma | p2 | HPV 18 |
| CC116 | 1 | Squamous cell carcinoma | P1 | HPV 16 |
| CC117 | 1 | Squamous cell carcinoma | P1 | HPV 16 |
| CC118 | 1 | Squamous cell carcinoma | P1 | HPV 16 |
| CC119 | <1 | Squamous cell carcinoma | P1 | HPV 16 |
| CC120 | <1 | Squamous cell carcinoma | P1 | HPV 16 |
| CC121 | <1 | Squamous cell carcinoma | p2 | HPV 18 |
| CC122 | <1 | Squamous cell carcinoma | p2 | HPV 18 |
| CC123 | <1 | Squamous cell carcinoma | P1 | HPV 16 |
| CC124 | 1 | Adenocarcinoma | P1 | HPV 16 |
| CC125 | 1 | Squamous cell carcinoma | negative | negative |
| CC126 | 1 | Squamous cell carcinoma | P1 | HPV 16 |
| CC127 | <1 | Squamous cell carcinoma | negative | negative |
| CC128 | <1 | Squamous cell carcinoma | p3 | HPV 31 HPV 35 |
| CC129 | <1 | Squamous cell carcinoma | P1 | HPV 16 |
| CC130 | <1 | Squamous cell carcinoma | P1 | HPV 16 |
| CC131 | <1 | Squamous cell carcinoma | P1 | HPV 16 |
| CC132 | <1 | Squamous cell carcinoma | p3 | HPV 31 |
| CC133 | <1 | Squamous cell carcinoma | P1 | HPV 16 |
| CC134 | <1 | Squamous cell carcinoma | negative | negative |

**P1: HPV 16**

**P2: HPV 18 and HPV 45**

**P3: HPV 31, HPV 33, HPV 35, HPV 52, and HPV 58**

**P4 : HPV 51 and HPV 59**

**P5 : HPV 39, HPV 56, HPV 66, and HPV 68**

**S1BTable: Diagnostic performance of the Xpert HPV test on formalin-fixed paraffin-embedded tissues of the cervix with reference to multiplex PCR.**

|  | **TP** | **TN** | **FP** | **FN** | **Sensitivity** | **Specificity** | **PPV** | **NPV** |
| --- | --- | --- | --- | --- | --- | --- | --- | --- |
| Xpert HPV | 118 | 12 | 0 | 1 | 99.16% | 100% | 100% | 92.31% |

TP: true positive, TN: true negative, FP: false positive, FN: false negative PPV: positive predictive value, NPV: negative predictive value
