# Supplementary material for: HPV testing in cervical cancer formalin-fixed paraffin embedded tissues: Reliability of the Xpert HPV test and high-risk HPV genotypes distribution in Tunisia
Source: PLoS One. 2025 Oct 24;20(10):e0333600. doi: 10.1371/journal.pone.0333600 (PMC12551861; doi:10.1371/journal.pone.0333600)
Supplement: S1 Fig — (PDF) [file pone.0333600.s002.pdf]

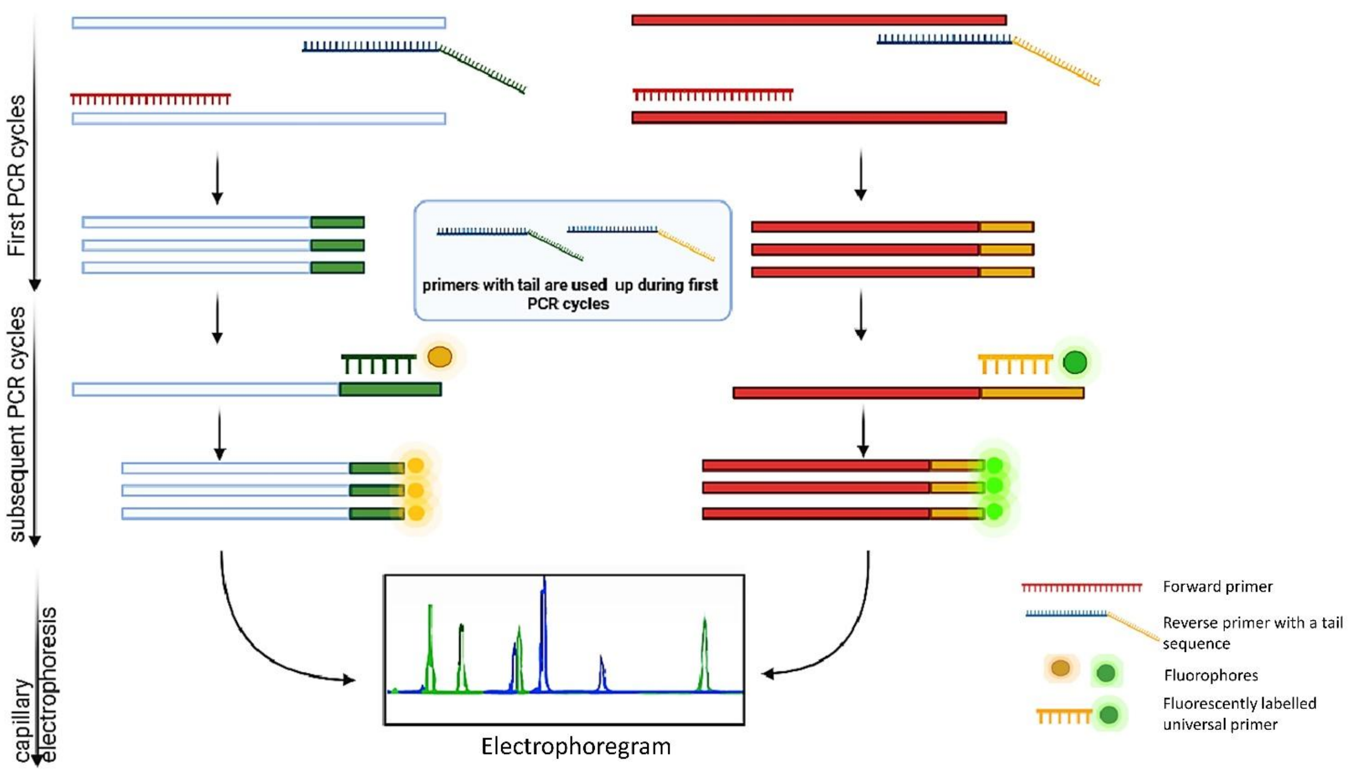

S2 Figure: Schematic representation of the triple-primed multiplex PCR strategy. This approach employs three primers for each target sequence: (i) a target-specific forward primer, (ii) a target-specific reverse primer carrying a non-complementary tail sequence at its 5' end, and (iii) a fluorescently labeled universal primer. The tail and universal primer sequences are identical and designed to avoid annealing to either the human or the viral genome. The reverse primer is added at a lower concentration, typically 10% of that of the forward and universal primers, to ensure efficient consumption during the initial PCR cycles. During the first PCR cycle, the complementary sequence of the tail is synthetized, and the reverse primers are used up, allowing subsequent amplification to be driven predominantly by the forward and universal primers. This results in a fluorescently labeled PCR product. For multiplex reactions, distinct tail sequences and different fluorescent labels are employed to differentiate between amplicons of close sizes.
